# Drug utilization pattern and adverse drug reactions of chemotherapy in pediatric patients at Muhimbili National Hospital, Tanzania

**DOI:** 10.12688/f1000research.110079.1

**Published:** 2022-04-06

**Authors:** Josephine Efraim, Castory Munisi, Auson Magige, Kelvin Msuya, Alphonce Ignace Marealle, Manase Kilonzi, Hamu Mlyuka, Wigilya Mikomangwa, Bertha Mallya, Wema Aswile, Kauke Bakari Zimbwe, Ritah Francis Mutagonda

**Affiliations:** 1 Department of Clinical Pharmacy and Pharmacology, Muhimbili University of Health and Allied Sciences, Dar-es-salaam, Tanzania, 0255, Tanzania; 2 Department of Pharmaceutics and Pharmacy Practice, Muhimbili University of Health and Allied Sciences, Dar-es-salaam, Tanzania, 0255, Tanzania; 3 Pharmacy department, Benjamin Mkapa Hospital, Dodoma, Tanzania, 0255, Tanzania

**Keywords:** Pediatric chemotherapy, Drug utilization pattern, Adverse Drug Reactions

## Abstract

**Background:** Cancer is a highly debilitating non-communicable disease and an essential contributor to the global burden of disease. Pediatric patients are highly exposed to multiple drugs for the management of cancer. Monitoring drug utilization patterns helps to provide feedback to healthcare providers to ensure the rational use of medicines; as a result, it increases the therapeutic efficacy and decreases the frequency and severity of adverse drug reactions (ADRs). Therefore, this study assessed the utilization pattern and ADRs of chemotherapy in pediatric patients at Muhimbili National Hospital (MNH).

**Methods:** A descriptive cross-sectional study was conducted for three months from February to April 2021 in pediatric cancer patients undergoing chemotherapy at MNH. A total of 123 children diagnosed with cancer and on chemotherapy were enrolled in this study. Patients’ socio-demographics, clinical information, chemotherapy status, prescribed medications, and prevalence of ADRs were collected. Descriptive statistics was used in data analysis, whereby frequency and proportions were used to summarize data.

**Results:** Out of 123 patients, 62.6% were male. Most patients received an average of four anticancer drugs. Vincristine (55.3%) was the most used anticancer drug, followed by cytarabine (44.7%) and methotrexate (42.3%). The most used adjuvant drugs were ondansetron (30.9%), hydrocortisone (27.6%), and piperacillin/tazobactam (23.6%). The percentage of drugs prescribed from the Tanzania Essential Medicine List (TEML) and World Health Organization (WHO) list was 66.4% and 93%. Most (87%) of the patients reported having experienced ADRs whereby nausea and vomiting (45.8%), hair loss (33.6%), and neutropenia (32.7%) were more prevalent ADRs reported.

**Conclusions:** This study found the drug prescribing pattern to be in line with the essential medicine list, but the average number of drugs prescribed was higher than recommended. ADRs were prevalent among pediatric cancer patients.

## Introduction

Cancer is a non-communicable disease (NCD) that reduces the quality of life. ^1^ It is a group of diseases that involves abnormal cell growth that invades or spreads to other parts of the body. ^2^ Cancer is a disease of public health importance, and it is reported to be among the leading causes of death globally in both developing and developed countries. ^3^ As per the World Health Organization (WHO) survey report, the global cancer incidence in 2012 increased to 14 million new cases. It is estimated that the incidence may rise to 19.3 million by 2025. ^4^ Worldwide, an estimated number of 250,000 children are diagnosed with cancer yearly, whereby most diagnoses occur in low and middle-income countries. ^5^

In Tanzania, the incidence of pediatric cancer is unknown due to the lack of a national cancer registry, but it has been estimated to be at 134 occurrences per million. ^6^ The likelihood of surviving a diagnosis of childhood cancer depends on the country; more than 80% of children with cancer are cured in high-income countries while in low-middle income countries only 30% are cured. ^7^^,^^8^ Cancer has contributed to 5.1% of all in-hospital deaths in Tanzania in 2006-2015. The mortality rate was 47.8 per 100000 population and the number of deaths was high among individuals 15-59 years of age. ^9^

Most used chemotherapy agents in cancer are cytotoxi *c*, meaning that they function by killing fast-dividing cells. The most immediate adverse drug reactions (ADRs) of chemotherapy are due to the cytotoxic effect on the normal cells. Cancer chemotherapy's common ADRs include hair loss, nausea and vomiting, anemia, febrile neutropenia, thrombocytopenia, tiredness, confusion, mood changes, tingling, burning, weakness, numbness, and pain in the hands and feet and mucositis. ^10^

The utilization pattern of anticancer drugs has changed significantly in recent years because of better enhancement in carcinomas' pathophysiology and the introduction of newer drugs. Significant inter-individual variability in the response rate of anticancer drugs, availability of different regimens, and combination regimens intolerability necessitate monitoring and evaluation of cancer chemotherapy.

Like many other low- and middle-income countries, the pediatric cancer outcomes in Tanzania are poor, and there are limited diagnostic and treatment capacities. ^5^ Moreover, it is unknown whether pediatric cancer patients are being managed rationally in Tanzania. Poor drug utilization among pediatric cancer patients will increase the occurrence of drug toxicity and ADRs hence decreasing the survival rates even further. ^11^ Therefore, this study assessed the drug utilization pattern and reported ADRs among pediatric cancer patients at Muhimbili National Hospital (MNH).

## Methods

### Study design

This hospital-based descriptive cross-sectional study was conducted from February to April 2021.

### Study area

The study was conducted at MNH which is the National Referral Hospital, a research center and university teaching hospital with 1,500 bed facility, attending 1,000 to 1,200 outpatients per day, admitting 1,000 to 1,200 inpatients per week. There are five government referral hospitals for dealing with cancer which are Muhimbili National Hospital, Ocean Road Cancer Institute, The Benjamin Mkapa Hospital, Mbeya Zonal Referral Hospital, Kilimanjaro Christian Medical Centre and Bugando Medical Centre which are mainly specialized in adult and pediatric malignancies. MNH has a special ward known as the Pediatric oncology ward that attends 60 to 70 pediatric cancer patients per month.

### Study population

The study was carried out on pediatric cancer patients admitted to the pediatric oncology ward and diagnosed with malignancy during the study period. The list of eligible participants was obtained from Tumaini and Upendo wards registers at MNH. These patients were then followed in their admission cubes whereby the parents or guardians who attend to them were told the details of the study. The consent was requested from parents followed by assent from children.

### Inclusion and exclusion criteria

All pediatric cancer patients receiving chemotherapy, aged less than 18 years old were included in the study. Exclusion criteria were patients whose diagnosis has not been well established and those with incomplete records in their files.

### Sample size and sampling

A total of 126 patients were enrolled in the study. The estimated sample size N was computed using Kish and Leslie formula given below:

$$N=\frac{Z^{2}\;P\;\left(100-P\right)}{\varepsilon^{2}}$$

N **=** estimated sample size

Z is percentage point of the normal distribution corresponding to the level of significance <5%,

Therefore, Z = 1.96.

P = Proportion of pediatric cancer patients on chemotherapy, from a study done in northern Tanzania on pediatric cancer patients, whereby a proportion of 93% was reported. ^5^

**ε** = margin of error, which is approximately 5%.

Systematic random sampling was used to select 126 patients out of the 240 patients. The IDs of 240 patients fulfilling the inclusion criteria were entered in Microsoft Excel followed by systematic random sampling whereby the sampling interval was 2.

### Data collection

A structured questionnaire was used to collect data. This tool was adapted from previous studies by *Bepari et al* and *Kamlekar et al* ^12^^,^^13^ with addition of demographic information to reflect the Tanzania context. The questionnaire consisted of socio-demographic information, clinical characteristics of the patients, drugs used, and reported ADRs. Patient socio-demographics included age, gender and residence. Clinical characteristics included the admission date, referral status, diagnosis, comorbidities, body mass index (BMI), hemoglobin levels. In addition, both anticancer, adjuvants drugs used and side effects at the time of data collection were recorded. The data collection tool can be found as *Extended data*. ^28^

### Data management and statistical analysis

Data collected was entered, cleaned, and analyzed using Statistical Package for Social Sciences ( SPSS, RRID:SCR_016479) version 24, and R statistical software version 4.0.3 (RRID:SCR_001905) was used for plotting. The data was summarized using frequency distribution and proportion. The continuous variables were summarized using median and interquartile range (IQR). The R scripts used in the analysis can be found as *Extended data.*

### Ethical considerations

Ethical clearance with reference number DA.25/11/01/dated 28 ^th^ January 2021 was obtained from the Muhimbili University of Health and Allied Sciences (MUHAS) Institutional Review Board (IRB). Permission to collect data from the hospital was obtained from the MNH administration. A signed informed consent was obtained from all parents/guardians before interview followed by assent obtained verbally from older children who were asked whether they would like to participate in the study and they said either a yes or no as an assent. For younger children 1-6 years which were majority of the study population, consent from the patient was enough for participation. Privacy and confidentiality were highly observed in data collection and person identifying information was not collected from the patient’s files.

## Results

### Baseline characteristics

**Figure 1.**
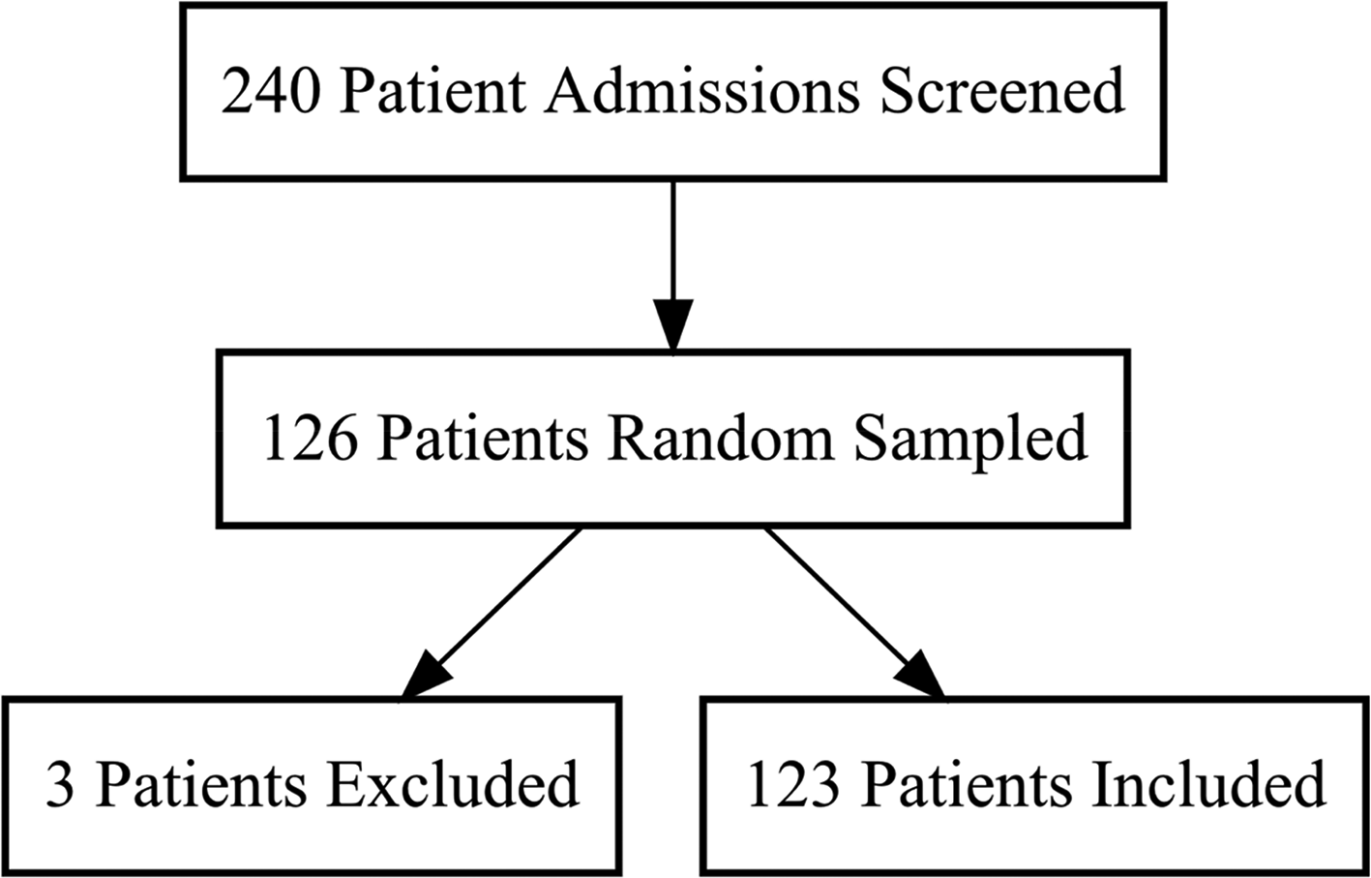
Study flow chart.

Overall, 240 patients were potentially eligible for the study. Systematic random sampling using Microsoft Excel was used to select 126 patients. Of the 126 patients, 123 were eligible and included in the final analysis and 3 patients were excluded. ^28^ Of the 3 excluded one refused to participate in the study and 2 had incomplete information in their files ( Figure 1).

Out of the 123 patients, the majority (62.6%, n = 77) were male. The median age was 5.2 (IQR = 6.4) years with half of patients (50.4%, n = 62) in the range of 0 – 5 years. The median weight was 16.7 (IQR = 10.4) kg, and almost half of the patients (48.8%, n = 60) were diagnosed in 2020. Based on hematological parameters, the median hemoglobin (Hb) level was 9.6 (IQR = 2.9) g/dl, median absolute neutrophil count (ANC) was 2.3 (IQR = 3.5) and the median platelet count (PLT) was 288 (IQR = 251). Most patients (79.3%, n = 98) were from regions outside of Dar es salaam ( Table 1).

**Table 1.** Demographic and clinical characteristics of the study patients (n = 123).

Characteristics	Number of patients (%)
**Residence**	
Dar es Salaam	25 (20.7)
Tanga	14 (11.6)
Pwani	10 (8.3)
Other regions	74 (59.4)
**Sex**	
Male	77 (62.6)
Female	23 (37.4)
**Age category**	
0 – 5 Years	62 (50.4)
6 – 10 Years	41 (33.3)
11 – 18 Years	20 (16.3)
**Diagnosis year**	
2018	1 (0.8)
2019	5 (4.1)
2020	60 (48.8)
2021	57 (46.3)
**Age in years** (Median, IQR)	5.2 (6.4)
**Weight in kg** (Median, IQR)	16.7 (10.4)
**Hb Levels in g/dl** (Median, IQR)	9.6 (2.9)
**ANC** (Median, IQR)	2.3 (3.5)
**PLT** (Median, IQR)	288 (251)

IQR Interquartile Range, Kg Kilogram, Hb Hemoglobin Levels, g/dl gram per deciliter, ANC Absolute Neutrophil Count, PLT Platelets.

Most patients (64.1%) had bone marrow and kidney malignancies with the prevalence of 30.1% (n = 37) and 27.6% (n = 34), respectively. The most dominant tumors were Wilms Tumor with 23.6% (n = 29) followed by B Cell Acute Lymphoblastic Leukemia (B Cell ALL) (17.1%) and Burkitt Lymphoma (17.1%) ( Table 2).

**Table 2.** Clinical characteristics of the study patients (n = 123).

Characteristic	Number of patients (%)
**Diagnosis**	
Wilms Tumor	29 (23.6)
B cell ALL	21 (17.1)
Burkitt Lymphoma	21 (17.1)
Retinoblastoma	18 (14.6)
T cell ALL	15 (12.2)
Hodgkin Lymphoma	12 (9.8)
Astrocytoma	4 (3.3)
Neuroblastoma	3 (2.4)
Ewing sarcoma	3 (2.4)
Xeroderma Pigmentosa	3 (2.4)
Rhabdomyosarcoma	2 (1.6)
Sacrococcygeal Teratoma	2 (1.6)
Squamous Cell Carcinoma	2 (1.6)
ALCL	1 (0.8)
Dysgerminoma	1 (0.8)
Hepatoblastoma	1 (0.8)
Nasopharyngeal Carcinoma	1 (0.8)
**Disease site**	
Bone Marrow (BM)	37 (30.1)
Kidney	34 (27.6)
Orbit	16 (13.0)
Neck	13 (10.6)
CNS	4 (3.3)
Jaw	5 (4.1)
Abdomen	1 (0.8)
Other (Bones, Genital, Muscles and Skin)	13 (10.6)

ALL Acute Lymphoblastic Leukemia, ALCL Anaplastic Large Cell Lymphoma.

### Drug utilization pattern

**Figure 2.**
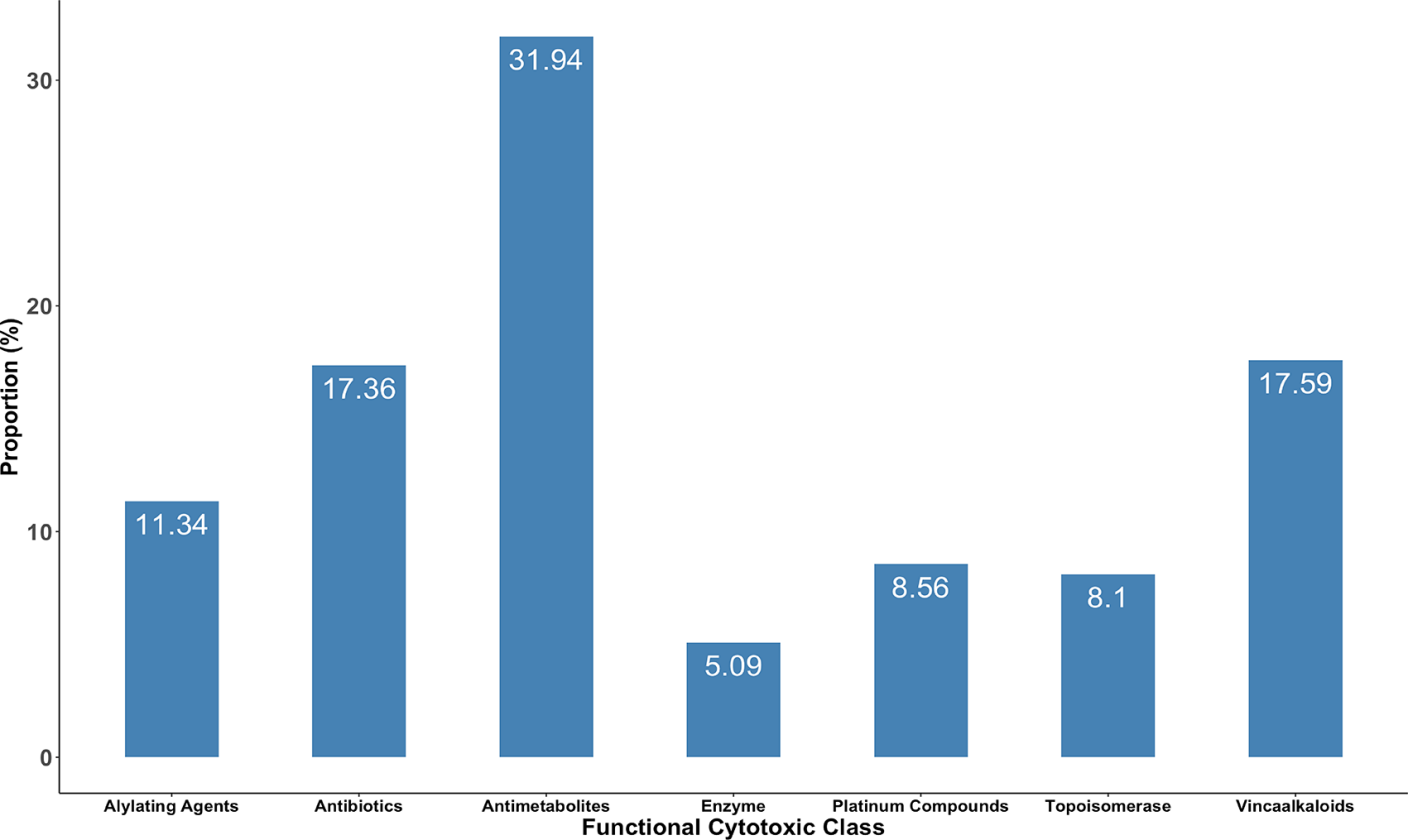
Cytotoxic drugs used in cancer treatment (n = 432).

The average number of drugs prescribed per prescription was 7 and the average number of cytotoxic drugs prescribed per prescription was 4. The percentage of drugs prescribed from the National Essential Medicines List (NEMLIT) and WHO Model Lists of Essential Medicines was 66.4% and 93%, respectively. More than a quarter (30.4%) of the prescribed drugs were injectables, 93.9% were prescribed using generic names and 19.0% of the medications were antibiotics. The most used class of anticancer agents were the antimetabolites (31.9%, n = 138) followed by vinca alkaloids (17.6%, n = 76) and antitumor antibiotics (17.4%, n = 75) ( Figure 2). Enzyme cytotoxic drugs were the least used 5.1% (n = 22). The commonly used anticancer drugs were Vincristine (55.3%, n = 68), followed by Cytarabine (44.7%, n = 55) and Methotrexate Injection (42.3%, n = 54) ( Table 3).

**Table 3.** Functional classification of chemotherapy medications (n = 432 prescriptions).

Functional classification	Frequency (%)	Chemotherapy medication	Frequency (%)
Alkylating agents	49 (11.3)	Cyclophosphamide	33 (26.83)
		Darcabazine	8 (6.50)
		Ifosfamide	8 (6.50)
Antitumor antibiotics	75 (17.4)	Doxorubicin	40 (32.52)
		Actinomycin D	15 (12.2)
		Bleomycin	10 (8.13)
		Actinomycin B	2 (1.63)
		Daunorubicin	8 (6.50)
Antimetabolites	109 (31.9)	Cytarabine	55 (44.71)
		Methotrexate	54 (42.28)
Enzyme	22 (5.1)	L-asparaginase	22 (17.89)
Platinum compounds	37 (8.6)	Carboplatin	30 (24.39)
		Cisplatin	7 (5.69)
Topoisomerase	35 (8.1)	Etoposide	35 (28.46)
Vinca alkaloids	76 (17.6)	Vincristine	68 (55.28)
		Vinblastine	8 (6.50)

Various adjuvants were given whereby the most commonly used adjuvants were antiemetic ondansetron injection (30.9%, n = 38), followed by steroids hydrocortisone injection (27.6%, n = 34) and cytoprotective agents such as dexrazoxane 24 (19.5%, n = 24) and mesna (21.9%, n = 27) ( Table 4).

**Table 4.** Functional classification of adjuvant medications (n = 432 prescriptions).

Functional classification	Adjuvant medication	Number of prescriptions (%)
Antibiotics	Piperacillin/tazobactam	29 (23.58)
Antiemetics	Ondansetron	38 (30.89)
Antihistamines	Cetirizine	14 (11.38)
Cytoprotective	Mesna	27 (21.95)
	Dexrazoxane	24 (19.51)
Laxatives	Lactulose	19 (15.45)
Proton Pump Inhibitor (PPI)	Pantoprazole	7 (5.69)
Steroids	Hydrocortisone	34 (27.64)
	Dexamethasone	12 (9.76)
	Prednisolone	6 (4.88)
Supplements	Folinic acid	7 (5.69)
Others	Benylin cough syrup, Maalox antacid syrup and Xylocaine local anaesthetic (BMX *)	11 (8.94)
	Furosemide	1 (0.81)

* Compounded in ratio of 4:4:1, Benylin cough syrup 100 mls, Maalox antacid syrup 100 mls and Xylocaine local anaesthetic 25 mls.

### Reported adverse drug reactions

**Figure 3.**
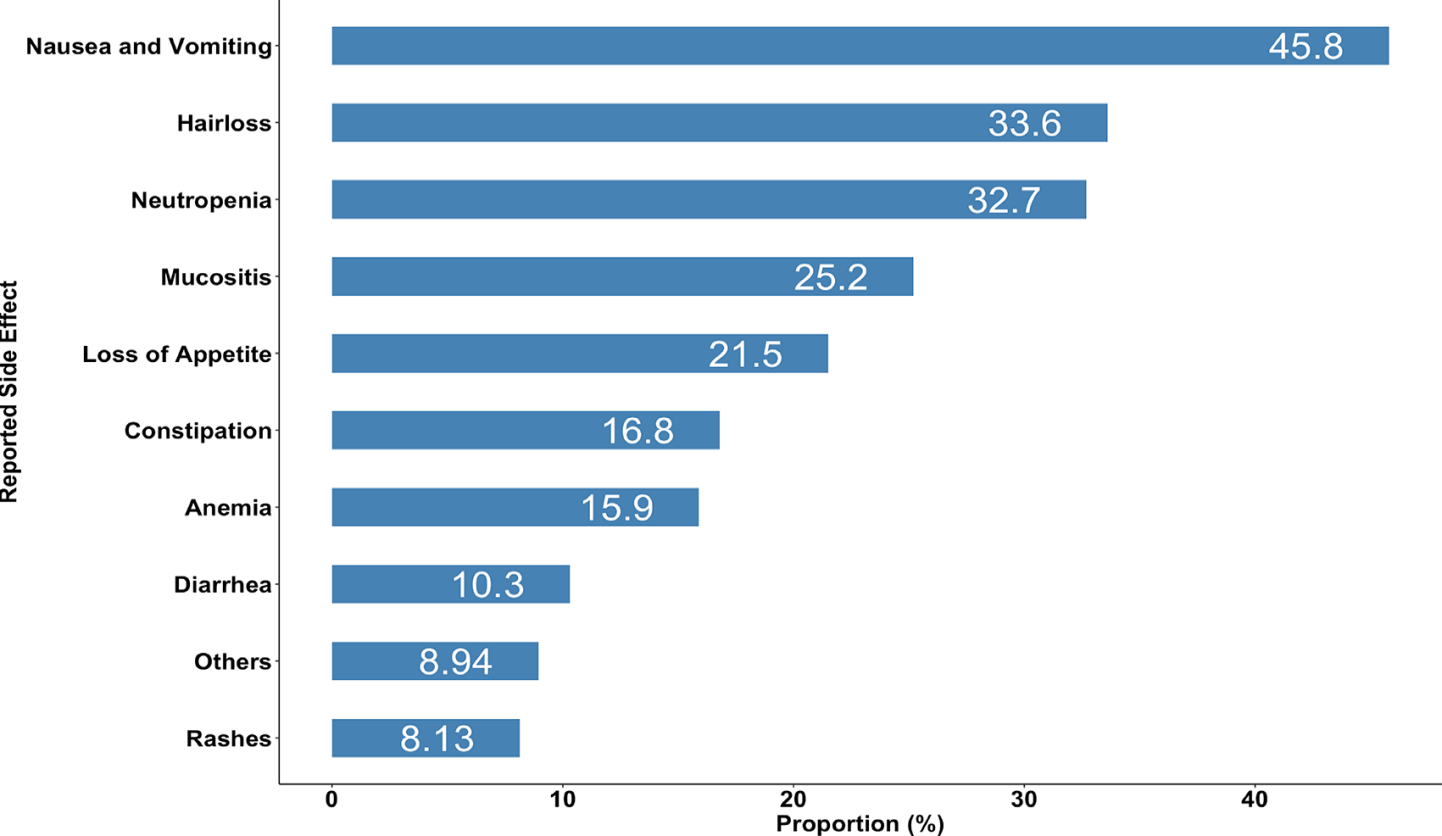
Side effects reported among pediatric cancer patients (n = 123).

Over three-quarters of the patients (87%) reported having experienced ADRs upon using chemotherapy medications. The most prevalent ADRs were nausea and vomiting reported by almost half of the study patients (45.8%) followed by hair loss and neutropenia with the prevalence of 33.6% and 32.7%, respectively ( Figure 3).

## Discussion

Assessment of drug utilization pattern is important as it provides information that will help in promoting the rational use of medication. Unlike the adult population, there is limited information on drug utilization pattern and ADRs experienced by pediatrics undergoing cancer chemotherapy in Tanzania. Therefore, this study assessed drug utilization pattern and reported ADRs of chemotherapy among pediatric cancer patients undergoing chemotherapy at MNH.

In this study the most predominant malignancies were Wilms Tumor, B Cell Acute Lymphoblastic Leukemia, Burkitt Lymphoma and Retinoblastoma. This is comparable to a study by Schroeder *et al*. which reported on the most prevalent pediatric cancers in northern Tanzania were Burkitt Lymphoma 18% and Wilms tumor 14%. ^5^ Similar malignancy types among pediatric patients have also been reported in other African countries. ^14^^,^^15^

The average number of drugs prescribed per prescription in this study was 7 which is higher than the WHO recommended range of 1.6 – 1.8. ^16^ This is comparable with the range of 6.0-6.9 which was reported in previous studies. ^12^^,^^13^ The average number of cytotoxic drugs prescribed per prescription was 3.5 which is higher than a study by Sandeep *et al*. and Bepari *et al*. in which it was 1.94 and 1.27, respectively. ^12^^,^^13^ This could be explained by differences in prescribing pattern from country to country influenced by existing cancer management guidelines, medicines availability, clinicians’ preferences, cost of medicines, diseased population, and disease status in the area. ^17^

The percentage of drugs prescribed from NEMLIT and WHO Model Essential Medicines List were 66.4% and 93.0%, respectively. The discrepancy observed in compliance to these two lists is attributed by the fact that the NEMLIT used during the study was not updated since 2017 compared to the WHO Essential Medicines List which has been updated in 2019. ^18^^,^^19^ After the study completion there was a release of updated NEMLIT in 2021 whereby most drugs have now been included in the management of cancer in pediatric patients.

In this study vincristine was the most used anticancer drug, followed by cytarabine and methotrexate which are both antimetabolites. Vincristine is the drug for most pediatric malignancies which was the target population in this study. ^20^ Similar findings were reported in Ethiopia whereby 85.4% of the pediatric patients were using vincristine. ^14^ The results differ from those obtained from the adult population in India in which carboplatin was the most prescribed drug, followed by paclitaxel and gemcitabine. ^12^ Also, they differ to those in another study conducted in India by Vijayalakshmi *et al*. on drug utilization pattern reported that cisplatin 58% and 5-fluorouracil 41% were most prescribed among all anticancer drugs, followed by doxorubicin. ^21^

In our study the most used adjuvant drugs were ondansetron, hydrocortisone and piperacillin/tazobactam. This is comparable to other studies in which similar adjuvants were also found to be commonly used for reduction of the ADRs of chemotherapy medications. ^12^^,^^13^^,^^22^

In the present study, over three-quarters (87%) of the study patients, reported to have experienced at least one side effect upon using chemotherapy medications. This is comparable to a study by Pearce *et al*. which looked at the incidence and severity of self-reported chemotherapy side effects in routine care in which 86% of the study patients reported at least one side effect during the study period. ^23^ The findings are also comparable to a cross-sectional national survey done in the U. S by Henry *et al*. in which 88% of the study patients reported at least one side effect. ^24^ Furthermore, a study done in Kenya by Opanga *et al*. looking at side effects of chemotherapy 93 % of the study reported to have experienced at least one side effect during chemotherapy treatment. ^25^

In our study the most prevalent side effects were nausea and vomiting, which were reported by about half of the study patients, followed by hair loss and neutropenia. This is comparable to the studies conducted in Malaysia and India, where the most common side effects of anticancer drugs included nausea and vomiting, hair loss, loss of appetite, and tiredness or weakness. ^26^^,^^27^ This could be explained by the fact that most anticancer drugs are associated with nausea and vomiting.

This study is limited in its scope to a single institution. However, MNH being the only national hospital has capacity in terms of human resources and technology required in management of cancer patients in the country. Therefore, information obtained from this center provide the best indicator of the drug utilization pattern and ADRs experienced by pediatric cancer patients in the country. Some important information was missing in the patients’ files and prescriptions like duration of treatment, height and weight of patients, hence limiting patients’ enrollment in the study. Moreover, factors influencing prescription patterns by the clinicians were not examined.

## Conclusions and recommendations

The prescribing pattern among the pediatric cancer patients at MNH was highly adherent to the WHO Model Essential Medicines List. However, the average number of drugs per prescription was very high. Vincristine was the most used anticancer drug and ondansetron was the most used adjuvant drug. The prevalence of side effects was very high indicating a need for improvement in prescribing for pediatric cancer patients to avoid ending up in irrational medicine use which could hinder the achievement of the treatment goals.

Since the average number per prescription was very high, we recommend multidisciplinary teamwork between prescribers and dispensers to reduce polypharmacy which could in turn can improve the management of pediatric cancer patients. Moreover, high prevalent of ADRs among these patients requires a vigilant ADR monitoring system to ensure early detection, management and reporting of ADRs experienced by pediatric cancer patients.

## Data availability

### Underlying data

Mendeley Data: Dataset for a cross-sectional study on “Drug Utilization Pattern and Adverse Drug Reactions of Chemotherapy in Pediatric Patients at Muhimbili National Hospital”. https://doi.org/10.17632/gjcyvs5nfx.3. ^28^

This project contains the following underlying data:

- paediatric.xlsx
- combined.xlsx

### Extended data

Mendeley Data: Dataset for a cross-sectional study on “Drug Utilization Pattern and Adverse Drug Reactions of Chemotherapy in Pediatric Patients at Muhimbili National Hospital”. https://doi.org/10.17632/gjcyvs5nfx.3. ^28^

This project contains the following extended data:

- analysis. R (analysis script)
- data_analysis.Rproj (analysis script)
- Data Collection Tool.docx
- Consent Forms English and Kiswahili Versions.docx

Data are available under the terms of the Creative Commons Attribution 4.0 International license (CC-BY 4.0).
