# Supplementary material for: Drug utilization pattern and adverse drug reactions of chemotherapy in pediatric patients at Muhimbili National Hospital, Tanzania
Source: F1000Res. 2022 Apr 6;11:396. [Version 1] doi: 10.12688/f1000research.110079.1 (PMC11053343; doi:10.12688/f1000research.110079.1)
Supplement: Peer review history

# Reviewer report: Reviewer response for version 1

**DOI:** 10.5256/f1000research.121654.r217377

**Published:** 2024-04-26

**Authors:** Paola Muggeo

**Affiliations:** 1 Department of Pediatric Oncology and Hematology, University Hospital of Policlinic, Bari, Italy

The Authors report data about childhood cancer in Tanzania and the use of antineoplastic drugs, together with the ADRs.

 The importance of this paper is to report incidence and outcome of childhood cancer in a developing Country, which is the first step to work for a better medical support and outcome. However the paper needs major adjustment to be improved.

 First of all, about methods. 

 Diagnosis: the authors report that the most frequent diagnosis is Wilms' tumor, which is a noteworthy result since it may depend on differences in genetic background, being this tumor a genetically influenced cancer. However diagnosis should be categorized according to international WHO classification. Moreover how is the diagnosis and staging of disease performed? This should be reported in the methods.

 The modality of drug association and delivery should be detailed. Are there any prescribed protocols to administer polychemotherapy? which is the basis to deliver polychemotherapy? usually standardized protocols should be used. If this is the case, they should be cited in the methods.

 Results: The authors conclude about irrational medicine use, however in the field of polychemotherapy detailed protocols guide the correct use of antitumor drugs based on mechanism of action, pharmacokinetics, and pharmacodynamics.

 Is the 30% survival rate caused by poor response to chemotherapy or to severe side effects (such as infectious complication or other severe side effects). In other words, is there any possibility to check the tumor response after chemotherapy?

 The paper needs major reorganization to offer a key of interpretation of an important and urgent problem in developing Countries.

Is the work clearly and accurately presented and does it cite the current literature?

Partly

If applicable, is the statistical analysis and its interpretation appropriate?

I cannot comment. A qualified statistician is required.

Are all the source data underlying the results available to ensure full reproducibility?

Partly

Is the study design appropriate and is the work technically sound?

Partly

Are the conclusions drawn adequately supported by the results?

No

Are sufficient details of methods and analysis provided to allow replication by others?

Partly

Reviewer Expertise:

pediatric hematolgy and oncology

I confirm that I have read this submission and believe that I have an appropriate level of expertise to confirm that it is of an acceptable scientific standard, however I have significant reservations, as outlined above.

# Reviewer report: Reviewer response for version 1

**DOI:** 10.5256/f1000research.121654.r261864

**Published:** 2024-04-13

**Authors:** Sidharth Totadri

**Affiliations:** 1 Department of Paediatric Haematology and Oncology, ST JOHN'S MEDICAL COLLEGE AND HOSPITAL, Bengaluru, Karnataka, India

**Drug utilization pattern and adverse drug reactions of chemotherapy in pediatric patients at Muhimbili National Hospital, Tanzania**

**General comments**

             The authors describe a single-center cross-sectional study of drug utilization and adverse reactions in children with cancer. Studies from regions such as the sub-Saharan are the stepping stones for capacity building in low- and middle-income countries. The authors can address the following comments.

1. Introduction: The first paragraph on cancer can focus more on childhood cancer rather than describing what cancer is. It would be good to mention the global initiative for childhood cancer from the World Health Organization to set the stage for a study on children being treated for cancer.
2. Results:

- It would be good to know what proportion of patients were underweight or malnourished rather than the median weight.
- The term bone marrow malignancy is vague as solid tumors like neuroblastoma and lymphomas can disseminate to marrow too. The term ‘leukemias’ may be better and also represent the disease site well.
- It is interesting to note that Wilms tumor surpassed B cell ALL in being the commonest malignancy. Do the authors have an explanation for this?
- Was any grading system like CTCAE used for adverse events? It would be good to know the proportion of severe or grade 3 or 4 toxicity.
- All scientific terms in results must be predefined in methods. For example, anemia and neutropenia must be defined with the cut-off used. Again, this reiterates the need to use a classification system like CTCAE or an indigenous system if used by the center, for all toxicity events.
- Since the study is aimed at evaluating drug utilization and capacity building there should be more details on the route of administration and doses. This is particularly relevant for drugs such as methotrexate which can be administered orally, intravenously, and intrathecally. Drugs such as methotrexate and cytarabine can be administered at high doses and low doses, and the corresponding vial strengths needed would be different.
- With this large sample size, one would like to know of treatment-related mortality during the study period. How many patients succumbed to adverse events of chemotherapy and what were the specific etiologies?
- The term ‘adjuvant therapy’ in oncology typically refers to the cytotoxic therapy that follows local therapy in a solid tumor. ‘Supportive care’ medication is an alternative term. Again, the authors must define what constitutes adjuvant therapy in the methods section, with a brief description of the center’s supportive care protocols. There is no mention of antimicrobial prophylaxis such as co-trimoxazole which would be an indispensable part of supportive care. The indication of steroids such as hydrocortisone must be mentioned, as they can be part of the chemotherapy regimen too.
- Figure 3: the side effects that comprise ‘others’ must be listed in the legend or in the main text. A singular unexpected or severe adverse event would be as important as a common adverse event.

      3.It would be interesting to see if there was an unavailability of any specific drug during the study period that led to a delay in therapy, or a need to modify the regimen to             exclude or replace the unavailable drug.

Is the work clearly and accurately presented and does it cite the current literature?

Yes

If applicable, is the statistical analysis and its interpretation appropriate?

Yes

Are all the source data underlying the results available to ensure full reproducibility?

Yes

Is the study design appropriate and is the work technically sound?

Yes

Are the conclusions drawn adequately supported by the results?

Yes

Are sufficient details of methods and analysis provided to allow replication by others?

Partly

Reviewer Expertise:

Pediatric Hematology Oncology practice in a low- and middle-income country, supportive care, thalassemia, common childhood cancers

I confirm that I have read this submission and believe that I have an appropriate level of expertise to confirm that it is of an acceptable scientific standard, however I have significant reservations, as outlined above.

# Reviewer report: Reviewer response for version 1

**DOI:** 10.5256/f1000research.121654.r136029

**Published:** 2022-05-05

**Authors:** Divya Subramonian

**Affiliations:** 1 Department of Pediatrics, Division of Hematology-Oncology, University of California, San Diego, CA, USA

In this article, the authors have addressed a critical and important issue by looking at drug utilization and adverse drug reactions in pediatric cancer patients. The authors have provided a clear and sound introduction covering relevant literature. The paper is well presented and could be used as a good tool for clinicians and researchers while prescribing drugs to pediatric patients.

**Methods:**

 Although the study was done only in one hospital, the sample size, distribution between male and female, and distribution of age group seems reasonable. The process of sample collection is clear with inclusion and exclusion criteria. However, it would be great if the authors can address this:

- Is there a reason for collecting data during the mentioned 3-month period (Feb-April 2021)? Is it possible to include/get data for a longer period of time?

**Results:**

 The tables are well presented, taking into consideration the demographic and clinical characteristics of the patients. The chemotherapy and adjuvant medications are well tabulated indicating the frequency of intake. The figures give a clear representation of the cytotoxic drugs used in the study pointing out the most commonly used ones. The most common side effects are also shown in Figure 3. All of this information can be used as a reference for physicians and researchers alike.

 Nevertheless, it would be nice to have a couple of things addressed/discussed.

- In Table 1, the number of males and females does not add up to 123. I believe the numbers 77 males and 23 females should be revisited.
- This is probably beyond the scope of the paper, but it would be nice to see the number or percentage of pediatric patients recovering from the disease given the adverse effects.

**Discussion:**

 As the clinical treatment of pediatric patients has changed significantly over the last few decades, this study points out the adverse effects the drugs have on patients.

- It would be useful if the authors can address how long the side effects last and discuss a little bit about the risk-benefit analysis for the most commonly used chemotherapy drugs.

It is alarming to note that the average number of drugs prescribed per prescription is 7, which is several times higher than recommended by WHO.

- Finally, do the authors have a hypothesis for this discrepancy and if that is true in other parts of Tanzania and the world? A little more insight on this would be a great eye-opener.

Is the work clearly and accurately presented and does it cite the current literature?

Yes

If applicable, is the statistical analysis and its interpretation appropriate?

I cannot comment. A qualified statistician is required.

Are all the source data underlying the results available to ensure full reproducibility?

Yes

Is the study design appropriate and is the work technically sound?

Yes

Are the conclusions drawn adequately supported by the results?

Yes

Are sufficient details of methods and analysis provided to allow replication by others?

Yes

Reviewer Expertise:

Pediatric Cancer - Neuroblastoma

I confirm that I have read this submission and believe that I have an appropriate level of expertise to confirm that it is of an acceptable scientific standard.
